# Trace Determination of Linear Alkylbenzene Sulfonates: Application in Artificially Polluted Soil—Carrots System

**DOI:** 10.1155/2009/404836

**Published:** 2009-06-14

**Authors:** Caroline Sablayrolles, Mireille Montréjaud-Vignoles, Jérôme Silvestre, Michel Treilhou

**Affiliations:** ^1^Laboratoire de Chimie Agro-Industrielle (LCA), Ecole Nationale Supèrieure des Ingénieurs en Arts Chimiques Et Technologiques (INP, ENSIACET), Université de Toulouse, 4 Allées Emile Monso, 31029 Toulouse, France; ^2^Laboratoire de Chimie Agro-Industrielle (LCA), Institut National de la Recherche Agronomique (INRA), 31029 Toulouse, France; ^3^Véolia Environnement, Direction de la Recherche, du Développement et de la Technologie, 36 rue de Liège, 75008 Paris, France; ^4^Laboratoire d'Ecologie Fonctionnelle, Ecole Nationale Supèrieure Agronomique de Toulouse (ENSAT), Avenue de l'Agrobiopole, BP 32607 Auzeville tolosane, 31326 Castanet-Tolosan, France; ^5^Laboratoire de Chimie et Biochimie des Interactions, Centre Universitaire Jean-François Champollion, Place de Verdun, 81012 Albi cedex 9, France

## Abstract

Surfactants are widely used in household and industrial products. The risk of incorporation of linear alkylbenzene sulfonates (LAS) from biosolids, wastewater, and fertilizers land application to the food chain is being assessed at present by the European Union. In the present work, a complete analytical method for LAS trace determination has been developed and successfully applied to LAS (C10–C13) uptake in carrot plants used as model. These carrots were grown in soil with the trace organics compounds added directly into the plant containers in pure substances form. LAS trace determination (*μ*g kg^−1^ dry matter) in carrots samples was achieved by Soxtec apparatus and high-performance liquid chromatography-fluorescence detection. The methodology developed provides LAS determination at low detection limits (5 *μ*g kg^−1^ dry matter) for carrot sample (2 g dry matter) with good recoveries rate (>90%). Transfer of LAS has been followed into the various parts of the carrot plant. LAS are generally found in the carrot leaves and percentage transfer remains very low (0.02%).

## 1. Introduction

**Figure 1:**
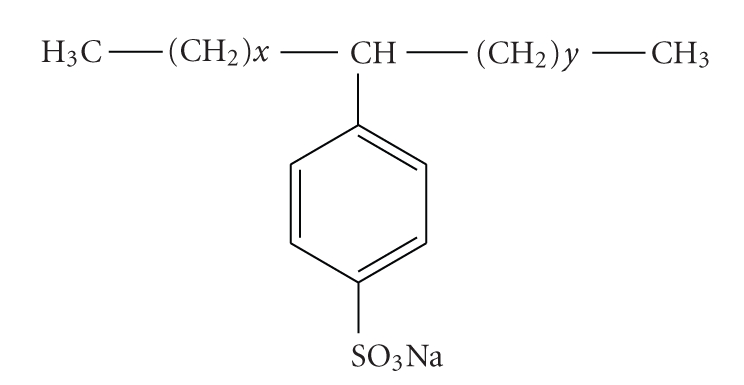
General chemical structure of linear alkylbenzene sulfonate (LASs), where x and y corresponds with the number of CH^2^ on each side of the benzene sulphonate group (7 • x + y • 10).

Linear alkylbenzene sulfonates (LASs) are synthetic anionic surfactants which were introduced in the 1960s as more biodegradable replacements for highly branched alkyl benzene sulfonates [1, 2]. LASs are nonvolatile compounds produced by alkylation and sulfonation of benzene [3]. LASs are a mixture of homologues and phenyl positional isomers, each containing an aromatic ring sulfonated at the para position and attached to a linear alkyl chain at any position except the terminal one (Figure 1). The product is generally used in detergents and cleaning products in the form of the sodium salt for domestic and industrial uses [2–4]. Commercially available products are very complex mixtures containing homologues with alkyl chains ranging from 10 to 13 carbon units (C10–C13). It corresponds to a compromise between cleaning capacity, on the one hand and biodegrading and toxicity, on the other hand. LASs have been extensively used for over 30 years with an established global consumption of 2 millions tons per year [5]*. *The properties of LASs differ greatly depending on the alkyl chain length and position on benzene sulfonate group. It has been found that longer LASs homologues have higher octanol/water partition coefficient (Kow) values [6, 7]. In fact, the homologues with long chain have a greater capacity of adsorption on the solids and a greater insolubility in the presence of calcium or magnesium [8]. In general, a decrease in alkyl chain length is accompanied by a decrease in toxicity [5]. Dermal contact is the first source of human exposure to LASs. Minor amounts of LASs may be ingested in drinking water, on utensils, and food. The daily intake of LASs via these media (exposure from direct and indirect skin contact as well as from inhalation and from oral route in drinking water and dishware) can be estimated to be about 4 *μ*g/kg body weight [9]. Occupational exposure to LASs may occur during the formulation of various products, but no chronic effects in humans have been noticed. In great concentration (500–2000 mg kg^−1^), LASs could have a long-term effect [8]. Indeed, their dispersing capacity could induce the release of others compounds present in soil [10]. Generated scrubbing could involve the biodisponibility of these new compounds [2]*.*

After use, LASs are discharged into wastewater treatment plants and dispersed into the environment through effluent discharge into surface waters and sludge disposal on lands. Moreover, LASs can be introduced directly into the grounds: their emulsifying and dispersing properties make them essential in the formulations of fertilizers and pesticides [11]. They are thus present in many compartments of the environment (sediments, aquatic environments, grounds…). LASs have been detected in raw sewage with a concentration range of 1–15 mg L^−1^ [9], in sludge with concentrations between 3–15 g kg^−1^ of dry matter [5, 8, 9], in surface waters at 2–47 *μ*gL^−1^ concentration range [9], and in soil at concentrations below 1 mg kg^−1^ [9, 10]. LASs can be degraded under aerobic conditions however are persistent under anaerobic conditions [9, 12]*. * Moreover, Lara-Martín and colleagues have shown that this surfactant can be degraded in sulfate-reducing environments such as marine sediments [13].

The determination of LASs in environmental samples is usually performed using liquid chromatographic methods with UV detection [4, 14, 15], fluorescence detection [16], or mass spectrometric detection [15, 17, 18] which allows the identification and determination of LASs isomers and homologues. There are a more limited number of gas chromatography methods [19, 20] which can be due to the low volatility of these compounds, being necessary the use of derivatisation reactions of the sulfonate group to obtain more volatile compounds. Capillary electrophoresis with UV detection has been also used for the determination of the sum, homologues and isomers of LASs in household products and wastewater samples [21]. Methods for the quantification of LASs in soil [18]*, *in sewage sludge [18, 19], in sediment [18, 22], in biological organisms [17, 23], or in water [14, 15, 20, 24] can be reported. However, these methods cannot be directly applied to plant analysis. Specific purification steps were needed. The main problem for analysis of organic pollutants in plants comes from the complexity of the matrix. Plants have a particular tissue structure, which depend on the species and the age, and are highly rich in pigments, essential oil, fatty acids, or alcohols. 

The risk of incorporation of LASs from biosolids, wastewater, and fertilizers land application to the food chain is being assessed at present by the European Union. The present study aims at developing and optimizing a method for LASs quantitative determination to detect their potential presence in food plants Carrots (*Daucus carota* L.) were studied because they provide a good maximizing uptake model for research. Indeed, carrots are root crops with high lipid content [25] and therefore LASs (amphiphilic nature) should easily be dissolved in these lipids. 

## 2. Experimental

### 2.1. Standards and Reagents

All chemicals used were analytical quality. Methanol, acetonitrile, and HPLC water were purchased from VWR Merck (France). Sodium perchlorate (NaClO_4_) and sodium dodecylsulfate (SDS) were obtained by VWR Prolabo (France).The cellulose extraction thimbles of 20 mm × 80 mm size were purchased from Schleicher & Schuell (France). Fontainebleau sand (particle size 150–300 *μ*m) to control boiling and powdered Florisil (Florisil PR particle size 60–100 mech-magnesium silicate) to adsorb grease were added to the sample in the cellulose extraction cartridge. The HPLC separation was performed with an Inertsil ODS3 column (C_18_) of 25 cm long × 0.46 cm internal diameter and 5 *μ*m particle size purchased from Supelco (France).

Condea Chimie SARL supplied the Marlon ARL which is a commercial surfactant powder containing 80% of C10–C13 LASs. This commercial homologue LASs mixture has the following homologue mass distribution: C10 (14.3%), C11 (35.7%), C12 (30.8%), and C13 (19.2%). Stock standard solution (1 g L^−1^) was prepared by dissolving 312.5 mg Marlon ARL in 250 mL methanol / SDS aqueous solution at 5 10^−3^ mol L^−1^ (50/50, v/v). 

### 2.2. Samples Collection

In order to test the different stages of the analytical protocol, carrots (*Daucus carrota *var. *Amsterdam A.B.K. Bejo*) were cultivated under greenhouse conditions (mean temperature 24°C, 14-hour light, 50% relative humidity). Fifteen days after germination, seedlings were transplanted into glass pots containing soil only (control pots) and soil artificially polluted with LASs (Marlon ARL): 1 g of Marlon ARL were mixed to 2 kg dry matter of soil. Four pots (2 L volume; 1510^−2^ m high; 13 10^−2^ m diameter) per treatment, each containing 7 carrots, were used. A soil sample was collected from the upper 20 cm of the field horizon from a French agricultural research station and was sieved at 5 mm. All plants were watered with nutrient solution (KNO_3_ 5,KH_2_PO_4_ 2, Ca(NO_3_)_2_ 5, MgSO_4_ 1.5 mmol L^−1^ for macronutrients, and Fe 15, Mn 0.49, Cu 0.06, Zn 0.11, Mo 0.01, B 0.26 mg L^−1^ for micronutrient) to 2/3 of field capacity in order to allow roots oxygenation. Carrots were harvested after 3 months and divided into leaves, peel, and core. Each part of the plants was carefully washed three times with demineralised water.

### 2.3. Sample Extraction

**Figure 2:**
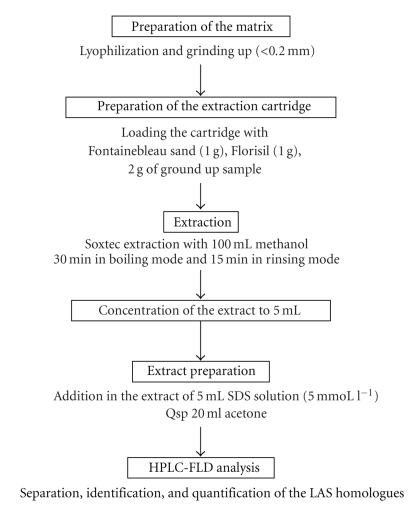
Description of the different treatment stages for LASs quantitative determination.

LASs determination in environmental samples was carried out according to a protocol of several determinative steps, that is, pretreatment, extraction, and analysis (Figure 2). Prior to extraction, samples were lyophilized and grounded up using a household grinder. This method allows recuperation of a maximum amount of trace organics.

The solid/liquid extraction was carried out with a Soxtec System HT2 (Tecator, France). This apparatus is a semiautomated apparatus working on the Soxhlet principle, while allowing extractions which are more rapid, economical (better solvent recuperation), and safe (dissociation of extraction and heating units). It is composed of two parts: an oil bath plus a unit with two plates heated by the oil, and above, systems for fixing the cartridge and for cooling. Two grams of lyophilized sample were extracted with 100 mL of methanol for 45 minutes. The sample was placed in a cellulose cartridge which was capped with cotton wool immersed in methanol (boiling mode) for 30 minutes to give a rapid, total contact. Next, the cartridge was lifted up above the still boiling solvent (rinsing mode) allowing the condensing solvent to rinse the sample. Then, a rotary evaporator (Rotavapor, Büchi) and 30°C temperature controlled bath was used to concentrate the solvent down to 10 mL. This extract is concentrated to 5 mL under a stream of nitrogen. 5 mL SDS solution (5 mmol L^−1^) and 10 mL acetone were added into the extract.

### 2.4. Sample Analysis

P200 (Spectra Physics) pump linked to a Hewlett Packard 1100 fluorimetric detection computer-controlled with data acquisition and processing using Normasoft (ICS) software, was employed. The system was equipped with an Inertsil ODS3 column and a guard column 2 cm long, 4.6 mm in diameter with a particle size of 5 *μ*m (Supelco, France). The volume injected (20 *μ*L) was reproducible and flow was 1.5 mL min^−1^ with the elution lasting for 35 minutes. The mobile phase was acetonitrile (A) and a premixed water/acetonitrile (75/25, v/v) solution containing sodium perchlorate (10 g L^−1^) (B) gradient programme (15%A–85%B at start, 2 minutes hold, 11 minutes linear gradient to 40%A–60%B, 1 minutes hold, 8 minutes linear gradient to 70%A–30%B and 5 minutes hold, 5 minutes linear gradient to 15%A–85%B and 3 v hold) and carried out at room temperature. The fluorescence detector operated at excitation-emission wavelengths of 225–305 nm. 

## 3. Results and Discussion

### 3.1. Extraction Time

Lyophilised carrots sample were spiked with LASs mixture (500 *μ*g kg^−1^ dry matter) just prior to extraction to test the effectiveness of the extraction procedure. This level has been chosen in order to be in coherence with the experimental culture. Three extraction times (45, 90, and 180 minutes) have been tested. Three replicates have been made for each extraction procedure. Recovery rate % and standard deviation for extraction times were found such as: 45 minutes : 91 ± 4; 90 minutes : 91 ± 9; 180 minutes : 89 ± 8. The results showed that there is no significant difference between the 3 extraction times. Thus, the total extraction time has been set at 45 minutes (30 minutes in boiling mode and 15 minutes in rinsing mode). 

### 3.2. Separation, Calibration Graphs, and Limits of Detection

**Figure 3:**
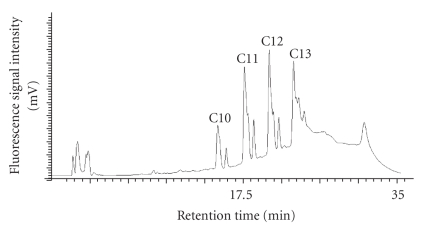
HPLC-FLD chromatogram obtained for carrot sample spiked with LASs (50 *μ*gg^−1^ dry matter). Chromatographic condition: Inertsil ODS3 colum of 250 × 4.6 mm (5 *μ*g), flow rate = 1.5 mL min^−1^, fluorescence detection (ex: 225 nm–em: 305 nm), 20 *μ*L of injection volume.

The homologues separation has been set up using reversed phase high-performance liquid chromatography coupled with fluorescence detector (HPLC-FLD) which has proved to be a successful method for determination of LASs in water and solid samples [14, 22, 23, 26, 27]*. *LASs separation by homologues is illustrated in Figure 3. This chromatograph corresponds to the analysis of a sample.

Calibration curves (equation: y = 3.64210^4^x + 4.30710^6^ ) were generated using linear regression analysis and over the established concentration range (5–100 *μ*gmL^−1^) gave good fits (*r* ^2^ > 0.990). LASs were identified by the retention time compared to the qualitative standard. The quantitative calculations are made from the peak area. LASs were determined as the sum of homologous C10 to C13 LASs. 

The precision of the method is given as the repeatability expressed as the relative standard deviation (R.S.D.%) and is an evaluation of the overall extraction - analysis procedure [28]. It is calculated from 5 replicates of 5 carrot samples. The R.S.D. was found to be 5% for the concentration level 30 mg LASs kg^−1^ dry matter.

The recoveries rate were found in the range of 91 ± 4% for carrot samples. The blank values, obtained from an empty sampler, were <5 *μ*g kg^−1^ dry matter.

The quantification limit has been based on 10 standard deviations (S.D.) of 10 replicates samples and was determined to be 25 *μ*g kg^−1^ of dry matter. The detection limit, based on 3 standard deviations (S.D.) of 10 replicates samples, was determined to be 5 *μ*g kg^−1^ of dry matter. This limit of detection is 100 times lower than those obtained by Gron et al. [27], and Mortensen et al. [26], for carrots analysis. 

### 3.3. Application

This method has been applied to carrots samples exposed to LASs with objective of evaluating potential bioconcentration of this surfactant. 

Initially carrots cultures on sand–LASs pure substances mixtures were carried out but carrots did not develop. Indeed, LASs induce a modification of the sand capillarity properties: water do not go up when it is introduced by the lower part of the farming system and water fall down when it is introduced by the higher part of the substrate. Thus, carrots cultures on soil with LASs pure substances were set up.

Statistical data processing were carried out after a variance analysis by the test with multiple degree of Newman-Keuls at *P* < .05 (Statistical Software, Sigma Stat 2.00). The same letter in a column means as there is no significant difference with *P* < .05. On the other hand, a different letter means that there is a significant difference between treatments.

Table 1 (line 1 and 2) presents the number of repetitions per treatment and the number of carrots per pot. In a general way, 7 carrots per pot were transplanted. With the stage of harvest, there do not remain inevitably these 7 carrots because of climatic and watering conditions. However, there is no significant difference between treatments regarding the number of carrots per pot. 

Dry matter production is presented in Table 1 (line 3). Newman-Keuls tests showed that there were no significant differences in growth between the carrots on soil only (control) and those on soil with LASs pure substances. It is thus clear that even in very great quantity, LASs do not inhibit the development of carrot under our experimental conditions. This result is in agreement with [27]. 

Average levels of LASs in core, peel, and leaves of carrot plants are presented in Table 1 (line 4). HPLC-FLD apparatus gives concentration results in mg L^−1^. Knowing sample mass and taking into account all analytical steps, results can be expressed in terms of mg kg^−1^ of dry matter. 

The percentage of transfer of LASs in carrot was calculated by submitting the ratio of the mass of LASs found in carrot on the mass of LASs present initially in the pot. Only, 0.02% of LASs initially spiked in soil have been uptake by carrot plants. Percentage repartition of LASs have been calculated by taking into account LASs flux in each carrot compartment and LASs total flux transferred in the three compartments. Mean of 12% LASs transferred are found in peel, 23% in core, and 66% in leaves. LASs are compounds likely to be transferred from the ground towards plant [27, 29, 30]. LASs properties responsible for this behavior are a great solubility in water without micelle formation and weak affinity for organic matter. Thus, LASs can be transferred towards the plants by water absorption by the roots. Indeed, LASs are surface-active anion which has the characteristic to have absorbent groups (sodium sulfonate) and hydrophobic groups (alkyl chain) (2). Thus, carrots are able to accumulate hydrophobic compounds [31] but the uptake observed in this experimentation remains very low.

## 4. Conclusions

An analytical protocol for LAS determination in carrots using Soxtec extraction (methanol, 30 minutes) and HPLC-FLD quantification has been developed. The methodology developed provides good recoveries rate (91 ± 4%), good precision (5%) and low detection limits (5 *μ*g kg^−1^ dry matter) for carrot sample (2 g dry matter). This method has been applied to the study of LAS bioavailability in carrots cultivated on soil enriched with pure trace organics. LAS have been traced in the various parts of the plant. LAS are generally found in carrot leaves. Plant analyses of LAS in carrots showed a weak plant uptake with LAS added as spike solution.

## Figures and Tables

**Table 1:** Number of carrots per pot; dry matter production; LASs concentration in soil, and LASs concentration in carrots. Mean values of four replications followed by the same letter in a column are not significantly different at *P* < .05, ± SE, standard error in variance analysis.

Line			Control carrots	LASs carrots
1	Number of pots	—	6	6
2	Number of carrots per pot	carrot	7.00 ± 0.50^b^	6.25 ± 0.48^b^
3	Dry matter production (mg per plant, mean ± S.E.)	Peel	82.5 ± 4.8^a^	85.0 ± 23.6^a^
		Core	188.0 ± 9.5^a^	197.0 ± 54.2^a^
		Leaves	210.0 ± 15.7^a^	233.0 ± 19.7^a^
4	Total content of LASs in soil (initial)(mg kg^−1^ dry matter, mean ± SE)	Soil	50 ± 4	500 ± 20
5	Total content of LASs in carrot plants(mg kg^−1^ dry matter, mean ± SE)	Peel	0.47 ± 0.04	0.63 ± 0.06
		Core	0.26 ± 0.03	0.31 ± 0.03
		Leaves	0.06 ± 0.01	0.26 ± 0.02
